# Influence of Fixation and Permeabilization on the Mass Density of Single Cells: A Surface Plasmon Resonance Imaging Study

**DOI:** 10.3389/fchem.2019.00588

**Published:** 2019-08-23

**Authors:** Ruoyu Cheng, Feng Zhang, Meng Li, Xiang Wo, Yu-Wen Su, Wei Wang

**Affiliations:** ^1^State Key Laboratory of Analytical Chemistry for Life Science, School of Chemistry and Chemical Engineering, Nanjing University, Nanjing, China; ^2^Department of Monoclonal Antibody Products, National Institutes for Food and Drug Control, Beijing, China; ^3^School of Pharmacy, Nanjing Medical University, Nanjing, China; ^4^Department of Clinical Pharmacology, Sir Run Run Hospital, Nanjing Medical University, Nanjing, China

**Keywords:** surface plasmon resonance imaging, fixation, permeabilization, immunofluorescence, osmotic pressure

## Abstract

Fixation and permeabilization of cells and tissues are essential processes in biological techniques like immunofluorescence and immunohistochemistry for cell biology studies. In typical procedures, the biological samples are treated by paraformaldehyde and Triton X-100 to achieve cellular fixation and permeabilization, respectively, prior to the incubation with specific antibodies. While it is well-known that the integrity of cell membrane has been broken during these processes, quantitative studies on the loss of cellular mass density and the enhancement of molecular accessibility at single cell level are still rare. In this study, we employed the surface plasmon resonance (SPR) imaging technique to monitor the mass density change of single cells during sequential fixation and permeabilization processes. We further utilize the osmotic responses of single cells to sugar molecules as an indicator to evaluate the integrity of cell membranes. It was found that, while fixation initially destructed the integrity of cell membranes and increased the permeability of intra- and extra-cellular molecules, it was permeabilization process that substantially induced significant loss in cellular mass density.

## Introduction

**Figure 1:**
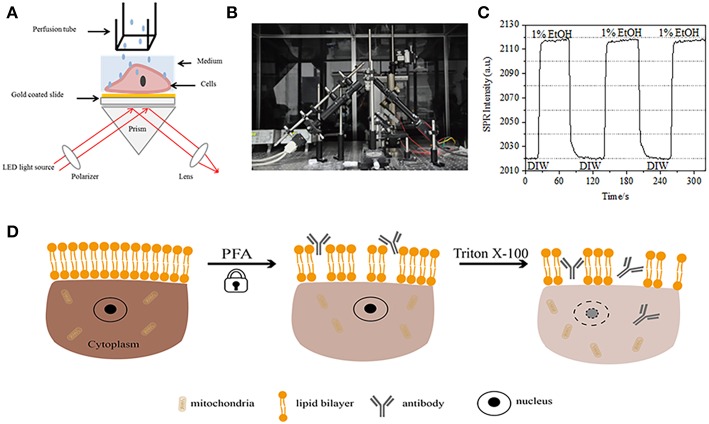
Prism-based SPR imaging setups. **(A)** Schematic illustration of the experimental set-up. **(B)** A photograph of the apparatus. **(C)** Sensitivity calibration curve. **(D)** Schematic illustration of cell fixation and permeabilization.

Immunofluorescence is a powerful technique to visualize the distribution of specific biomolecules within biological samples such as cells and tissues (Joshi and Yu, 2017). In typical cell-based immunofluorescent assays, adherent cells were incubated with fluorescent antibody to enable specific recognition and binding between the antibody and the target molecule in the cells. After thorough rinse, the sample was placed under fluorescence microscope to obtain a fluorescence image, from which the distribution of target molecules was reported by the fluorescent tags. In order to facilitate the accessibility of antibody to the target molecules and to inhibit the inherent cellular activity, the samples were often fixed and permeabilized prior to the staining procedures. They were particularly necessary when the target molecules were located within the cytoplasm. Among many types of reagents, paraformaldehyde (PFA) and Triton X-100 are probably the most widely used ones for fixation and permeabilization, respectively. Depolymerization of PFA produced formaldehyde molecules to create covalent chemical bonds between proteins in the sample. The mechanism of the action relied on the activation of one of the amino acid residuals lysine. PFA also dissolved some lipids in cellular membranes which slightly damaged the cell membrane integrity (Fox et al., 1985; Thavarajah et al., 2012; Kiernan, 2018). Triton X-100, as an effective non-ionic detergent, could dissolve lipids from cell membranes, so that the cell membrane became more permeable to the fluorescent antibody. The permeabilization step removed more cellular membrane lipids due to its uncharged, hydrophilic head groups that consist of polyoxyethylene moieties to allow large molecules like antibodies to get inside the cell (Jamur and Oliver, 2010; Koley and Bard, 2010). A schematic illustration of cell fixation and permeabilization process is shown in Figure 1D. Despite of the fact that fixation and permeabilization have become routine procedures in immunofluorescence and immunohistochemistry, pretty rare efforts have been made to quantitatively clarify their influences on the membrane integrity and cellular mass density at single cell level.

Surface plasmon resonance (SPR) has been a popular and powerful technique to determine the binding kinetics between a pair of molecules since its invention in 1980s (Liedberg et al., 1983; Cullen et al., 1987). This is attributed to its remarkable advantages, including real-time quantitative kinetic measurement with high temporal resolutions, compatibility with expanded devices, and most importantly, its intrinsic feature of label-free (Rothenhäusler and Knoll, 1988; Phillips and Cheng, 2007; Homola, 2008; Abadian et al., 2014; Méjard et al., 2014; Yanase et al., 2014; Su and Wang, 2018). Early applications mostly focused on characterizing and quantifying biomolecular interactions by immobilizing purified molecules onto SPR sensing substrates (gold-coated glass slides). Such *ex situ* studies not only required the labor-intensive purification procedures, but also led to results that may not reflect the natural interaction in living beings (Phillips and Cheng, 2007; Homola, 2008). Driven by both the technical advancement in various SPR imaging (SPRi) systems and the scientific motivation in single cell imaging and analysis, both prism and objective-based SPRi techniques have been employed to image the mass distribution of single living cells without the need of labeling (Wang et al., 2010, 2012a; Yanase et al., 2010; Yang et al., 2015; Zhang et al., 2015). Each one has its own merits. The latter has a higher spatial resolutions and the former has a better sensitivity. By monitoring the time-lapsed SPR images of single living cells during various types of physiological and biological stimulations, important spatial and dynamic information regarding the cell-substrate interactions (Giebel et al., 1999), cell migrations (Smith et al., 2004), osmotic responses (Wang et al., 2012a), ligand-receptor binding kinetics (Wang et al., 2012b, 2014), signaling pathways (Deng et al., 2016), protein activation dynamics (Peng et al., 2018), and living cancer cell drug responses (Wang et al., 2018) have been obtained. For instances, existing studies have clearly demonstrated that SPRi techniques were capable for mapping the mass density as well as the membrane integrity at single cell level (Yanase et al., 2010; Wang et al., 2012b; Yang et al., 2015). The image contrast of SPRi came from the subcellular distribution of refractive index, which was roughly determined by the local mass density. Binding of specific antibodies onto the cell membrane that expressed the corresponding antigens, or stimulating the living cells with particular chemicals, was found to alter the mass density of single cells in a heterogeneous and dynamic manner (Wang et al., 2012a,b). Exposure of single living cells to hypertonic solutions was found to induce the contraction of cells, indicating the excellent integrity of cell membranes (Wang et al., 2012a). However, how the fixation and permeabilization treatments would affect the SPR images of single cells remains unclear.

In the present work, we employed a home-built SPRi system to continuously record the time-lapsed SPR images of tens of single living cells when the cells were successively treated by 4% PFA solution and 1% Triton X-100 solution. The mass density of single cells was determined by the averaged SPRi signal. The membrane integrity was evaluated by exposing the cells to hypertonic solution. It was found that, in addition to the slightly reduced mass density by <10%, PFA treatment significantly destructed the cell integrity as indicated by the loss of osmotic response upon the exposure of sugar molecules. Subsequent treatment by Triton X-100, however, significantly reduced the mass density by another 20%, suggesting the severe destruction to the membrane integrity.

## Materials and Methods

### Materials

Dulbecco's phosphate buffered saline (PBS, Gibco), Sucrose (Sinopharm Chemical Reagent Co., Ltd), Triton X-100 (Aladdin), Paraformaldehyde (Shanghai Lingfeng Chemical Reagent Co., Ltd). All of the reagents were dissolved in PBS.

### Cell Culture

BT-474 cells were cultured at 37°C with 5% CO_2_ and 70% relative humidity in Dubelco's Modified Eagle's Medium (DMEM, Invitrogen) with 10% fetal bovine serum (FBS, Invitrogen), 100 units/mL penicillin and 100 μg/mL streptomycin (Invitrogen). Cells was passed when they were 70–80% confluent by treating with 0.25% trypsin solution (Gibco).

### Prism-Based SPR Imaging Setups

A schematic diagram and a photo of the SPRi system are presented in Figures 1A,B. The SPRi apparatus is mainly composed of three parts: light source, optical components and camera.

- Light source: A 670 nm light-emitting diode (L7868-01, Hamamatsu, Japan) and a temperature-controlled mounting socket (LDM21, Thorlabs, Newton, USA).
- Optical components (Edmund Optics, USA): A triangle SF-11 prism with a sensor chip on (No. 1 BK7 glass from Fisherbrand), a converging lens, a polarizer, and a tunable lens.
- Camera: Charge-coupled device camera (Pike F032B, Allied Vision Technology, Newburyport, USA).

The p-polarized light hits the bottom of the gold-coated glass slide through the prism directly and the camera captures the reflected beam to generate SPR images at a rate of 0.828 Frame per second (fps). When choosing 4X magnification in a tunable lens, the field of view was ~2 × 2 mm^2^.

### Sensor Chip Preparation

Each chip was washed with 75% ethanol and deionized water (DIW), followed by UV exposure for 30 min to remove the surface contamination and sterilize them before each experiment. A Flexi-Perm silicon chamber (Greiner Bio-One) was placed on top of the gold chip to serve as a cell culture well. To achieve the surface modification prior to cell seeding, a 100 μg/mL 150 μL collagen solution was added to the chamber and kept in an incubator for 2 h. The chip was then rinsed with deionized water twice and DMEM twice prior to cell seeding. To make the cells a good shape, the chip was incubated in the growth medium for 36 h. The growth medium was then replaced with PBS buffer solution for 10 min for cells achieving a balance.

### Flow System

A gravity driven multichannel drug perfusion system was applied to injection and switch of the solution. The flow rate is 300 μL/min and the solution switching rate around the cells can reach 1–2 s.

### Sensitivity Calibration

A sensor chip was prepared as described in preceding part without surface modification and cell seeding. First, DIW flowed over the chip for about 60 s. Then, it was replaced by 1% (v/v) ethanol solution for another 60 s. As one of the most commonly used calibration solution, 1% ethanol is known to increase the SPR angle of DIW by 60 mDeg. Experimental results showed that SPR intensity accordingly increased by 96 I.U. (Figure 1C), corresponding to a sensitivity of 1.6 ΔI.U./mDeg.

## Results and Discussion

The cell membrane (also known as cytoplasmic membrane), consisting of a lipid bilayer with embedded proteins, is a semi-permeable membrane that encloses the cytoplasm of a cell. The fixation and permeabilization steps of cells and tissue samples, which could alter the permeability of cell membrane, are crucial procedures that could determine the successes of immunofluorescent or immunohistochemical assays. In addition to this, the antibody quality and the immunoreaction procedure are other key determinants to these kinds of experiments. SPR has the feature that both the resonant angle and the refractive index (RI) near the sensing surface are highly sensitive to the mass density of the surface layer in the medium-metal interface. Therefore, cell's mass density variations taking place on or near the metal film (~200 nm) could be synchronously recorded by measuring the intensity of the reflected light. By employing surface plasmon resonance imaging technique, we explored dynamic distribution of cellular mass density with high spatial and temporal resolutions and reliable sensitivity in both fixation and permeabilization processes in this study.

### Initial Mass Density Loss in Cell Fixation by PFA

**Figure 2:**
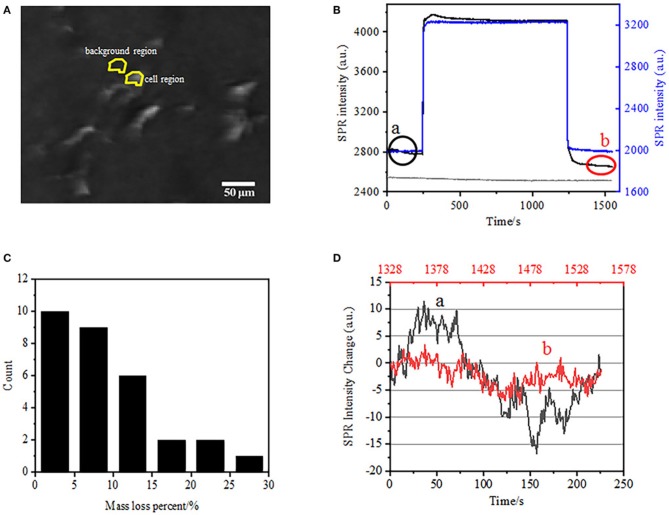
Cell fixation process. **(A)** SPRi image of BT-474 cells. **(B)** SPR signals of single cell (black curve) and background (blue curve) undergoing a solution switching process: PBS buffer to 4% PFA to PBS buffer. SPR signal of single cell treated with PBS during 1,500 s (gray curve). **(C)** Mass loss distribution of 30 single cells. **(D)** Noise level of the cell before (a) and after (b) PFA treatment.

The first step to prepare biological samples for immunofluorescent or immunohistochemical analysis is usually fixation. And the most commonly used fixatives for these kinds of assays are crosslinking fixatives like PFA that works by generating covalent chemical bonds between proteins in cells or tissues. We therefore investigated the influence of PFA treatment on cellular mass density. Living BT-474 cells were cultured on coverslip coated by gold film with a thickness of 50 nm. A representative SPR image containing single cells is provided in Figure 2A. SPR signals of each cell can be obtained by choosing a region-of-interest in the SPR image that matches the morphology of the investigated cell. In a typical experiment, when solution flowed over the cells, SPR sensorgrams of the cells were obtained by analyzing each cell as shown in Figure 2B (black curve). In the preceding 242 s, PBS buffer without PFA was flowing over the cells and the baseline was determined by the system stability (light source and mechanical stability) and the inherent micromotion signals generated by the living cells. PFA solution was then introduced at the 242th second, which immediately increased the SPR signal because of the relatively large refractive index of 4% PFA solution (bulk effect). PFA solution ran for another 17 min to achieve sufficient fixation. At the 1237th second, PFA solution was switched back to pure PBS solution. In addition to the reduced bulk effect, a decrease in the SPR intensity of single cells was found when comparing the signals before (0–242) and after (1237–1545) the PFA treatments and the mass loss percent was obtained through dividing the decrease value to the initial value. The SPR sensongrams of the background (an adjacent region without cell adhesion, blue curve) and a single cell treated with PBS during 1,500 s (gray curve) are also shown in Figure 2B. Statistical analysis on 30 cells reveals an averaged mass density loss of 10 ± 5% as shown in Figure 2C. It was also found that, after PFA treatment, the baseline fluctuation level in SPR signal was significantly reduced by half or even two third, suggesting the loss of micromotions and fixation of the treated cells as shown in Figure 2D. Such micromotion signals were generated by a bunch of dynamic cellular activities from different cell components including skeleton, membranes and organelles. The fixation terminated such biological activities and therefore eliminated the micromotion signals, accompanying with a significantly reduced intensity fluctuation in the SPR sensograms of single cells.

### Osmotic Pressure Response in Cell Fixation by PFA

**Figure 3:**
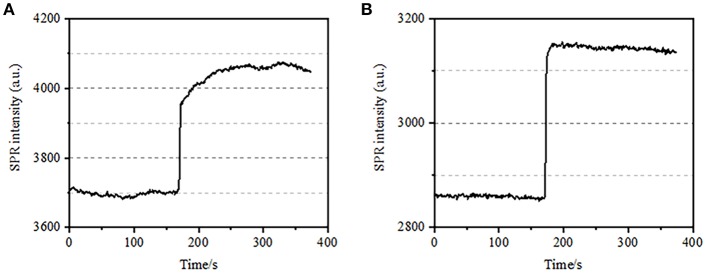
Osmotic pressure responses of cells. **(A)** Response of a living cell to 25 mM sucrose. **(B)** Response of the same cell fixed (10 min) to 25 mM sucrose.

The integrated structure of cell membrane, a semi-permeable membrane, regulates the homeostasis of a cell to reach balance in physiological osmotic pressure. PFA treatment was also found to have the ability in destructing the membrane integrity as shown in Figure 3. When the buffer around the target cell changed into 25 mM sucrose solution in <1 s via a drug-perfusion system, a hypertonic cell culture environment was created. For living cells, the exposure to hypertonic solution triggered a series of physiological responses to balance the intracellular and extracellular osmotic pressure, which has been investigated in details in our previous work (Wang et al., 2012a). In this study, a typical SPR sensorgram of single physiological BT-474 cells is provided in Figure 3A. Upon the exposure to hypertonic sucrose solution, treated cells firstly underwent a pressing shrink, leading to a gradual increase in SPR intensity until a plateau was reached. It suggested the increased mass density as a result of cell shrink and subcellular components gathering toward the bottom of the gold culture coverslip. The gradual increase in SPR intensity by hypertonic stimulation is a kind of physiological regulation for cellular osmotic pressure, thus indicating the physiological integrity of the cell membrane. When the membrane integrity was destroyed, the introduction of hypertonic sucrose solution was found to immediately increase the SPR intensity in a much shorter time, as shown in Figure 3B. It is because the sugar molecules freely penetrated into the cells without resistance from the semi-permeable membrane and increased the local refractive index immediately (bulk effect). It is clear that, while the slow increase in the SPR intensity reflected the physiological osmotic regulation (Figure 3A), the rapid jump demonstrated the cell membrane had been damaged to allow for the free entry of small sugar molecules (Figure 3B).

### Substantial Mass Density Loss in Cell Permeabilization by Triton

**Figure 4:**
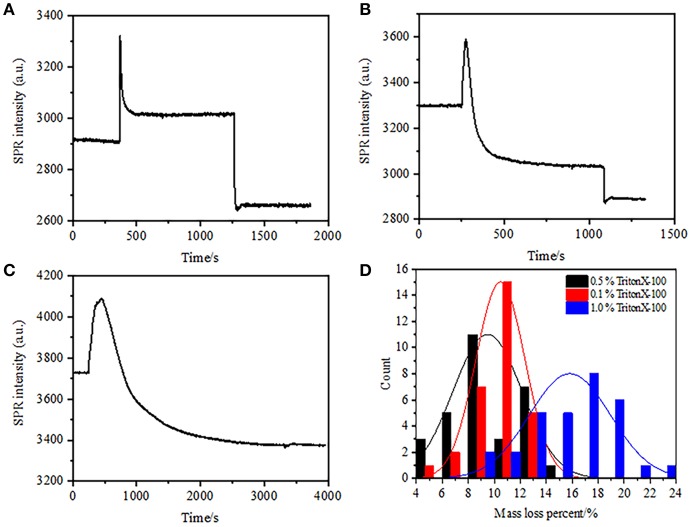
Mass loss curves of cell treated with different concentration solutions **(A)** 1.0%, **(B)** 0.5%, **(C)** 0.1%. **(D)** Mass loss distribution of 30 single cells in different conditions.

In the previous fixation stage, an initial cell mass density decreased just by ~10% (Figure 2C). It is reasonable to speculate that the PFA fixation only slightly destructed the membrane integrity and increased the permeability of small molecules. Majority of the intracellular components should be still harbored within the cytoplasmic membrane. We subsequently studied the influence of a typical permeabilization detergent, 1% Triton X-100, on cells that have been fixed with PFA for 17 min previously. A representative SPR sensorgram of single cells during exposure to 1% Triton X-100 is shown in Figure 4A. The introduction of Triton X-100 increased the SPR intensity followed by a gradual decrease. The first increase is a consequence of increase refractive index of bulk solution, and the followed decrease indicated the gradual loss in the mass density due to the cell permeabilization. The reduced mass density was more plentiful when comparing the SPR intensity change before (0-300 s) and after the introduction of detergent (1,300–1,800 s). Analysis on 30 single cells demonstrated that the Triton X-100 treatment created a substantial mass density decrease by another 20 ± 5% after PFA fixation as shown in Figure 4D and Table 1. It is clear that the detergent Triton X-100 eliminated much more cellular membrane lipids and severely destructed the membrane integrity in this permeabilization stage. This resulted in more intracellular components, especially macromolecules, to release from the cells. And also, this allows large molecules like antibodies to get inside the cell in the next immunoreaction stage. Although SPR intensity of small molecules leaching was detected after PFA fixation, permeabilization by using Triton X-100 detergent clearly solubilized lipids and made the cells much more permeable to the movements of macromolecules in and out of the cell body.

**Table 1:** A summary of variations in mass density loss of statistic cells in different conditions.

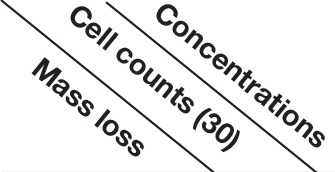	**1.0 %**	**0.5 %**	**0.1 %**
	**Triton X-100**	**Triton X-100**	**Triton X-100**
4–6%	0	3	1
6–8%	0	5	2
8–10%	2	11	7
10–12%	2	3	15
12–14%	5	7	5
14–16%	5	1	0
16–18%	8	0	0
18–20%	6	0	0
20–22%	1	0	0
22–24%	1	0	0

It was found that the loss in mass density is a rather rapid process, which occurred in a few minutes in permeabilization stage (Figure 4A). The decrease in mass density roughly followed a monotonic decay within a time period of 126 s. The typical sensorgrams of single cells under 0.5 and 0.1% Triton X-100 are displayed in Figures 4B,C, respectively. The permeabilization process became much slower when reducing the concentration of detergent. It took 126, 510, 2,600 s for the three kinds of the treated cells to finish mass loss.

## Conclusion

In summary, dynamic redistribution of cellular mass density in both fixation and permeabilization processes have been explored sensitively by using prism-based SPRi setup with high spatial and temporal resolutions. Fixative PFA could initially increase the permeability of cytoplasmic membrane to some extent because its ability of fixing cellular proteins in both membrane and intra-cellular proteins. It was supported by (1) the decreased mass density by ~10% after a 17-min fixation treatment in 4% PFA solution, and (2) the eliminated cellular response to hypertonic solution, which demonstrated the destruction of cellular integrity to small molecules like sugars. Detergent Triton X-100 is superior to fixative PFA in solubilizing lipids and therefore increasing cell membrane permeability, accompanying with the more substantial loss in the mass density due to the release of not only small molecules but also large molecules and possibly some organelles such as vesicles. Besides, the rate of mass loss is positively correlated with Triton X-100 concentration. These results provided quantitative and dynamic understandings on the influence of fixation and permeabilization on the cellular mass density and membrane integrity, with implications for optimizing the conditions for single cell biological experiments, such as immunofluorescent and immunohistochemical assays.

## Author Contributions

RC, Y-WS, and WW designed the experiments. RC, FZ, ML, and XW performed the experiments. RC, Y-WS, and WW wrote the paper. RC, FZ, Y-WS, and WW discussed the results and analyzed the data.

### Conflict of Interest Statement

The authors declare that the research was conducted in the absence of any commercial or financial relationships that could be construed as a potential conflict of interest. The handling editor declared a shared affiliation, though no other collaboration, with the authors WW, RC, ML, and XW at time of review.
